# Gastrointestinal Complications of Systemic Sclerosis: A Case Report

**DOI:** 10.7759/cureus.86844

**Published:** 2025-06-27

**Authors:** Mason Arbabi, Bella Garg, Saviz Saghari, Paryus Patel

**Affiliations:** 1 Internal Medicine, University of Kentucky College of Medicine, Lexington, USA; 2 Internal Medicine, Centinela Hospital Medical Center, Inglewood, USA; 3 Internal Medicine, West Anaheim Medical Center, Anaheim, USA; 4 Internal Medicine/Pulmonology, Centinela Hospital Medical Center, Inglewood, USA

**Keywords:** gastroparesis, hospice and palliative care, limited systemic sclerosis, progressive dysphagia, small intestinal bacterial overgrowth

## Abstract

Systemic sclerosis (SSc) is a rare, chronic systemic rheumatic disease. Gastrointestinal (GI) involvement is highly prevalent in SSc and any part of the GI tract may be affected. This can range from mild symptoms like heartburn and dysphagia to severe issues like abdominal distention, malnutrition, and fecal incontinence. In SSc patients, GI tract dysfunction significantly impacts the quality of life and is a major contributor to both morbidity and mortality. This case report highlights an 80-year-old female patient with SSc/CREST (Calcinosis, Raynaud's phenomenon, Esophageal dysfunction, Sclerodactyly, and Telangiectasia) syndrome experiencing severe GI involvement who presented to the emergency department with hypoxic respiratory failure and acute kidney injury. The patient and her family finally made the decision to transition to inpatient hospice care, focusing on comfort and dignity, leading to the patient's peaceful passing.

## Introduction

Systemic sclerosis (SSc) is a chronic multisystemic disease characterized by autoimmunity, vascular dysfunction, and fibrosis of the skin and internal organs [1]. Nearly 90 percent of patients with SSc have evidence of gastrointestinal (GI) involvement including oropharyngeal, esophageal, gastric, small intestinal, colorectal, hepatic, and pancreatic [2]. SSc can cause a wide range of GI complications, affecting various parts of the digestive tract from the mouth to the rectum: oropharyngeal dysphagia, esophageal motility disorder, gastroesophageal reflux, gastroparesis, gastric antral vascular ectasia (GAVE), small intestinal bacterial overgrowth (SIBO), hypomotility intestinal pseudo-obstruction, malnutrition, colonic inertia, diarrhea, and fecal incontinence [3]. GI symptoms can cause increased morbidity and include dysphagia, choking, heartburn, hoarseness, cough, early satiety, bloating, alternating diarrhea with constipation, malabsorption, and fecal incontinence [4]. Early recognition and management of GI issues in SSc patients are crucial and can significantly impact their long-term outcomes and quality of life.

## Case presentation

**Figure 1:**
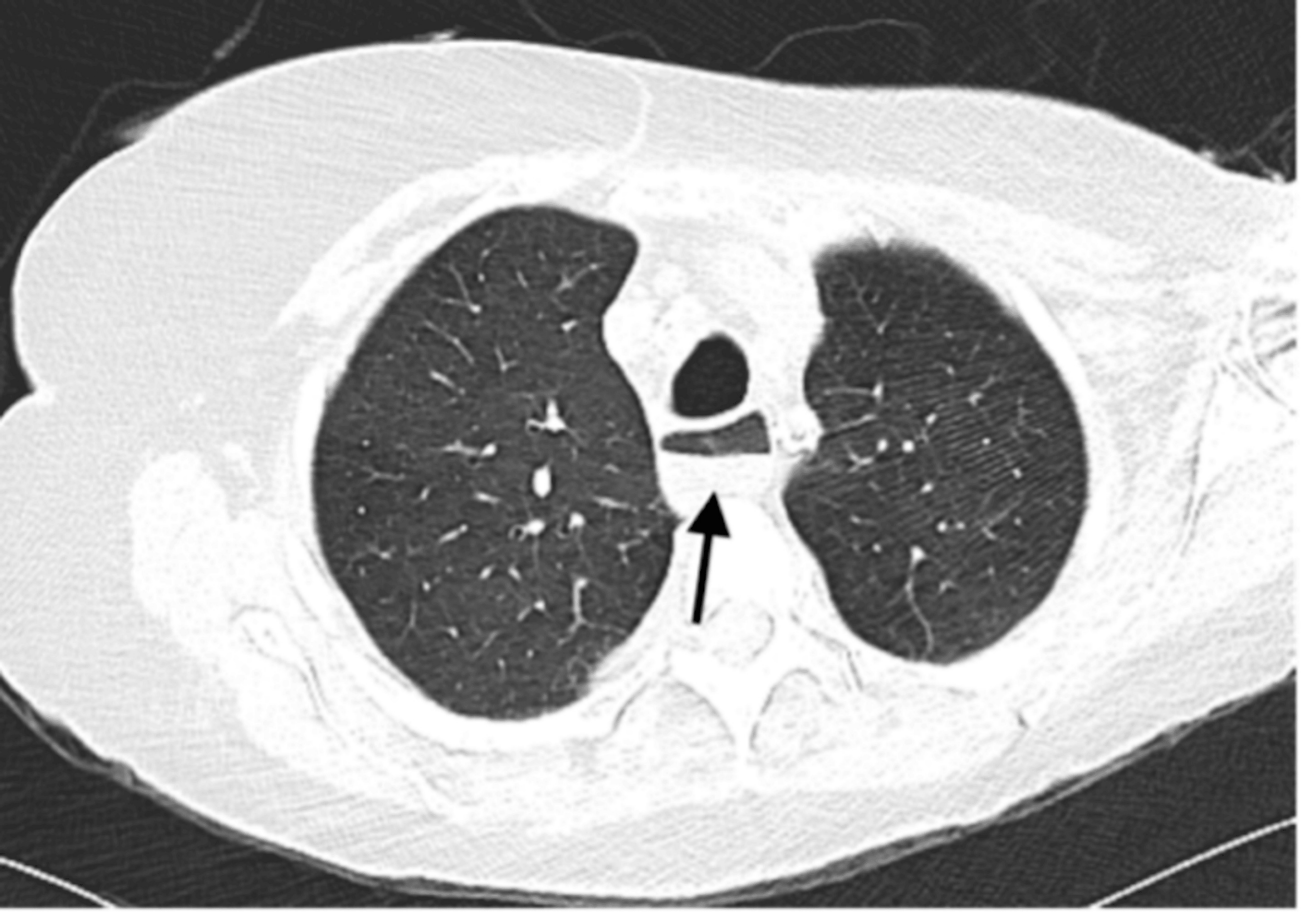
Patulous and fluid-filled esophagus (arrow) increasing the risk for aspiration.

This case highlights an 80-year-old woman with a history of limited cutaneous SSc, heart failure with preserved ejection fraction, pulmonary hypertension, obstructive sleep apnea, diabetes mellitus, hypertension, dyslipidemia, bilateral carotid stenosis, cerebrovascular accident/stroke, chronic kidney disease (CKD), peripheral artery disease, and hypothyroidism who presented in November to the emergency department with acute on chronic hypoxic respiratory failure and acute kidney injury on CKD. Over the past few months, she also experienced progressively worsening symptoms of severe dysphagia, choking, heartburn, nausea, vomiting, early satiety, diarrhea, abdominal pain, and fecal incontinence. Her home medications to manage the GI symptoms include omeprazole 40 mg, simethicone 80 mg, ondansetron 4 mg, loperamide 2 mg, polyethylene glycol, docusate sodium, and Florajen probiotics. Significant findings on physical examination included obesity, bibasilar crackles with decreased breath sounds on auscultation, and +2 pitting edema bilaterally on the lower extremities. Additionally, she had features of incomplete CREST (Calcinosis, Raynaud's phenomenon, Esophageal dysfunction, Sclerodactyly, and Telangiectasia) syndrome with clinical evidence of Raynaud’s phenomenon, esophageal dysmotility, sclerodactyly along with edema distal to the metacarpophalangeal joints, and telangiectasia with no evidence of calcinosis. The laboratory evaluation displayed the presence of a strongly positive ANA (antinuclear antibody) of 1:1280 with an anti-centromere pattern, a marginally positive anti-double-stranded DNA of 1:160, negative anti-Smith antibody, negative anti-RNP (ribonucleoprotein) antibody, low positive RF (rheumatoid factor) of 36 IU, and negative anti-CCP (cyclic citrullinated peptide) antibody. Chest CT angiography showed no pulmonary embolism, increased bilateral pleural effusions, moderate-sized pericardial effusion, cardiomegaly, bilateral atelectasis, and patulous and fluid-filled esophagus (Figure 1).

**Figure 2:**
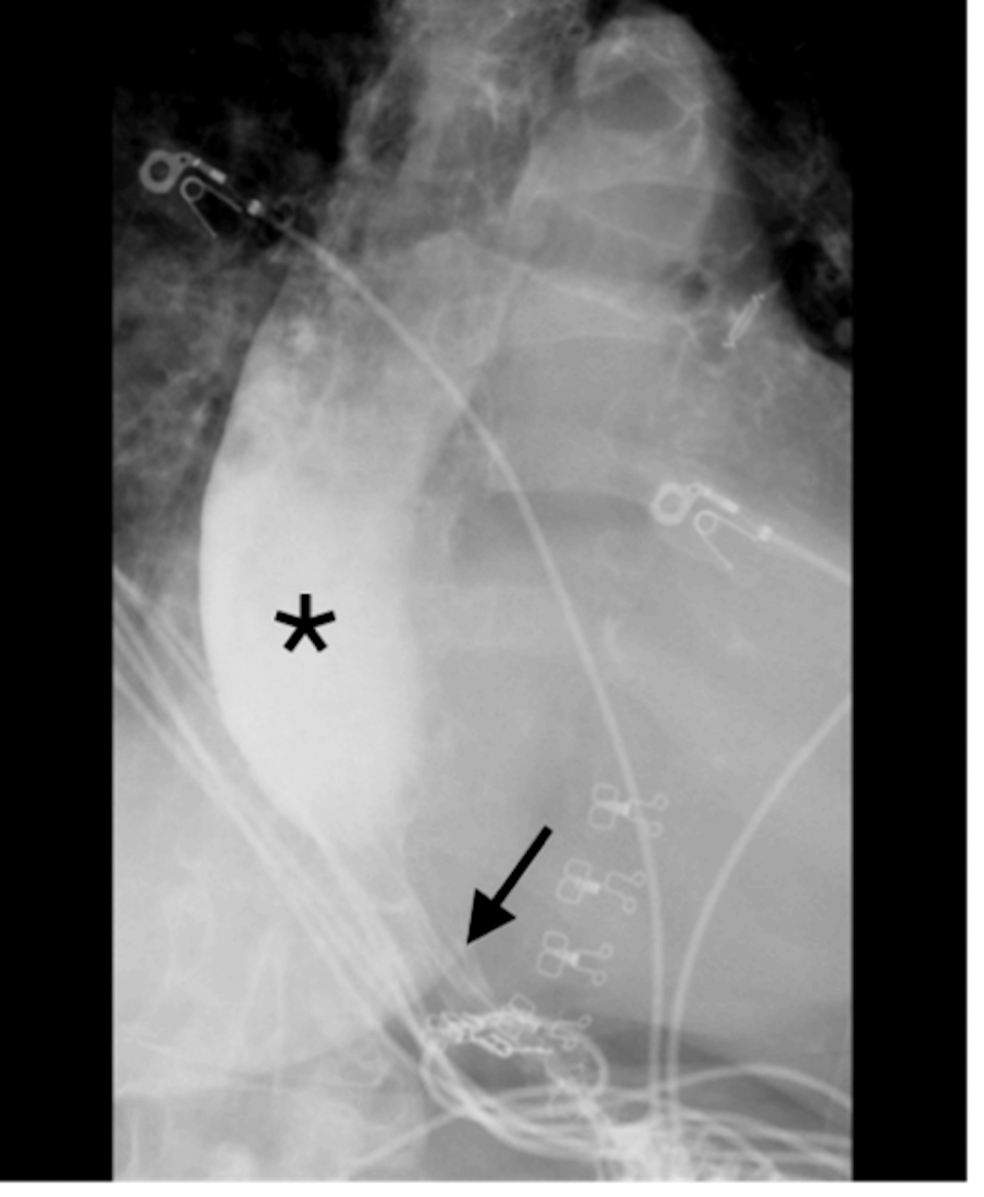
Dilated esophagus (star) with severe dysmotility. Long-segment smooth narrowing (arrow) of the distal gastroesophageal (GE) junction with very minimal contrast seen to transverse across the GE junction.

The pulmonary function test showed stable FVC (forced vital capacity) at 79% and DLCO (diffusing capacity of the lungs for carbon monoxide) at 76%. Echocardiogram was consistent with moderate pulmonary hypertension, right ventricular systolic pressure between 55 and 60 mmHg, and grade 2 diastolic dysfunction. In order to identify and diagnose structural and functional problems, a Barium swallow test was performed utilizing video fluoroscopy in conjunction with the Speech Pathology team. The test showed a dilated esophagus with frank dysmotility. The lower two-thirds of the esophagus demonstrated no peristalsis. Additionally, it showed severe narrowing just proximal to the gastroesophageal junction, resulting in delayed transit of contrast from the distal esophagus into the stomach explaining her severe dysphagia symptoms (Figure 2).

GI, rheumatology, cardiology, pulmonology, and nephrology services were consulted for further evaluation. Subsequently, the patient was placed on high-dose intravenous bumetanide. She continued to have minimal urine output, and her respiratory symptoms did not show any signs of improvement. Moreover, her renal function also deteriorated rapidly, ultimately necessitating scheduled hemodialysis, which she declined. The GI service opted against performing an upper endoscopy, given her high risk of anesthesia-related complications. A trial of rifaximin was also attempted for a possible diagnosis of SIBO, resulting in mild improvement of her diarrhea. Finally, after a long discussion with the patient and her family, a unified decision was made to transition her to inpatient hospice care, where she eventually passed away peacefully.

## Discussion

Oropharyngeal dysphagia occurs in up to 25 percent of patients with SSc [5]. Involvement of the oral and perioral skin and soft tissues can lead to significant complications, including microstomia, periodontitis, xerostomia, and impaired swallowing, resulting in oral food leakage, retention, and aspiration [6]. In SSc, smooth muscle atrophy in the distal two-thirds of the esophagus and the lower esophageal sphincter (LES) contributes to esophageal dysfunction, affecting 50 to 80 percent of patients [7,8]. An incompetent LES can result in gastroesophageal reflux, increasing the risk of reflux esophagitis and, if left untreated, subsequent esophageal stricture formation. Upper endoscopy may reveal findings such as reflux esophagitis, infectious esophagitis (e.g., candidiasis), Barrett's esophagus, and esophageal stricture. Esophageal manometry typically depicts a hypotensive LES with a low resting sphincter pressure (<10 mmHg) and low-amplitude (<30 mmHg) contractions in the distal smooth muscle segment of the esophagus, or aperistalsis. Additionally, a barium esophagogram, as it was done in our case, in patients with SSc may present an air-filled esophagus, impaired or absent peristalsis, esophageal dilation, and a rapid flow of contrast in the upright position; this feature is often described as a "waterfall esophagus" [9]. The most common gastric manifestation is gastroparesis (delayed gastric emptying), while GAVE, also known as "watermelon stomach", is a less frequent but notable complication [10]. Her severe GI symptoms including reflux, nausea, vomiting, early satiety, bloating, and abdominal pain could be indicative of gastroparesis, possibly stemming from SSc and/or diabetes. Severe gastroparesis may lead to intractable nausea and vomiting, potentially causing weight loss and nutritional deficiencies. The small intestine is the second most commonly affected site in the GI tract, with manifestations arising from impaired peristalsis that predisposes to stasis and intestinal dilatation [11]. A potential complication, SIBO, refers to an imbalance of gut microorganisms often resulting from intestinal stasis and pooling of food and waste, creating an environment conducive to bacterial overgrowth. Bloating is the most common symptom in SIBO, although patients may experience flatulence, abdominal discomfort, and diarrhea, which can lead to malabsorption and subsequent malnutrition [12]. Our patient's positive response to rifaximin, particularly with improvement in diarrhea, is indeed suggestive of SIBO as a possible underlying cause of her symptoms. Patients with hypomotility and ineffective peristalsis often report recurrent or chronic abdominal pain and bloating, while those with intestinal pseudo-obstruction may present more acutely, with symptoms such as abdominal pain, nausea, vomiting, and marked abdominal distension [13]. Colonic disease affects up to 50 percent of patients with SSc, most frequently involving the anorectum, and presenting with symptoms such as constipation, diarrhea, or fecal incontinence [14]. Diarrhea in SSc is often multifactorial, resulting from SIBO, pancreatic exocrine insufficiency, or overflow from constipation [15]. Anorectal involvement may lead to fecal incontinence and, sometimes, rectal prolapse. It can also be noted that the presence of diarrhea can further contribute to fecal incontinence in SSc patients including our specific case [16].

## Conclusions

The GI manifestations of SSc such as gastroesophageal reflux, choking, abdominal pain, bloating, diarrhea, constipation, and fecal incontinence can profoundly impact a person's quality of life, causing significant discomfort and disrupting daily activities. In severe cases, these complications may be debilitating to a person's well-being. This case illustrates the clinical course and end-of-life/hospice consideration for a patient with systemic sclerosis/CREST syndrome suffering from multiple severe GI symptoms.
